# Modulation of Plasma Membrane Composition and Microdomain Organization Impairs Heat Shock Protein Expression in B16-F10 Mouse Melanoma Cells

**DOI:** 10.3390/cells9040951

**Published:** 2020-04-12

**Authors:** Tim Crul, Balint Csoboz, Imre Gombos, Annamaria Marton, Maria Peter, Gabor Balogh, Csaba Vizler, Lajos Szente, Laszlo Vigh

**Affiliations:** 1 Institute of Biochemistry, Biological Research Centre, Szeged 6726, Hungary; 2 Institute of Medial Biology, University of Tromsø, Tromsø 9037, Norway; 3 Cyclolab Cyclodextrin R&D Laboratory Ltd., 1097 Budapest, Hungary

**Keywords:** plasma membrane, lipid raft, caveolae, HSP70, HSP25, cyclodextrin, nystatin

## Abstract

The heat shock response (HSR) regulates induction of stress/heat shock proteins (HSPs) to preserve proteostasis during cellular stress. Earlier, our group established that the plasma membrane (PM) acts as a sensor and regulator of HSR through changes in its microdomain organization. PM microdomains such as lipid rafts, dynamic nanoscale assemblies enriched in cholesterol and sphingomyelin, and caveolae, cholesterol-rich PM invaginations, constitute clustering platforms for proteins functional in signaling cascades. Here, we aimed to compare the effect of cyclodextrin (MβCD)- and nystatin-induced cholesterol modulations on stress-activated expression of the representative HSPs, HSP70, and HSP25 in mouse B16-F10 melanoma cells. Depletion of cholesterol levels with MβCD impaired the heat-inducibility of both HSP70 and HSP25. Sequestration of cholesterol with nystatin impaired the heat-inducibility of HSP25 but not of HSP70. Imaging fluorescent correlation spectroscopy marked a modulated lateral diffusion constant of fluorescently labelled cholesterol in PM during cholesterol deprived conditions. Lipidomics analysis upon MβCD treatment revealed, next to cholesterol reductions, decreased lysophosphatidylcholine and phosphatidic acid levels. These data not only highlight the involvement of PM integrity in HSR but also suggest that altered dynamics of specific cholesterol pools could represent a mechanism to fine tune HSP expression.

## 1. Introduction

When exposed to stress, cells induce the heat shock response (HSR), a multi-level signaling network characterized by the accumulation of a conserved set of so-called stress/heat shock proteins (HSPs) [1]. Being chaperoning proteins, HSPs recognize and prevent non-native protein conformations from forming deleterious protein aggregates during stress and, once the stressful event passed, assist in refolding or proteasomal degradation, depending on the extent of harmful exposure [2]. Based on structural similarities, HSPs are classified in several groups, including HSPA (HSP70), HSPB (small HSPs), HSPC (HSP90), HSPH (HSP110), HSPD/E (HSP60/HSP10), DnaJB (HSP40), and CCT (TRiC) [3]. Tight regulatory control of HSP expression is exerted by heat shock factor-1 (HSF1) which, under physiological conditions, mainly resides in the cytosol as an inactive monomer in complex with multiple HSPs. Stress-induced titration of HSPs from HSF1 allows it to quickly adopt a trimeric conformation, which is able to move into the nucleus while being modulated by multiple posttranslational modifications [4].

Based on this original model, HSP induction was primarily thought to be activated by protein denaturation and aggregation. However, it is now recognized that cells sense heat stress and activate the HSP expression machinery in multiple ways. For example, exposure to elevated temperatures fluidizes the plasma membrane and alters its physical properties and microdomain organization [5]. This activates fluidity-associated feed-back mechanisms controlling stress-responsive genes including HSPs [6]. Moreover, by acting as membrane-stabilizing factors [7], temporary association of certain HSPs with the plasma membrane [8] reduces its fluidity level [7], elevates bilayer stability [9], and thus restores membrane functionality during heat stress. Intriguingly, chemically induced PM fluidization and lipid raft reorganization with benzyl alcohol to levels similar as what is generally observed under heat stress caused a downshift of the HSR threshold resulting in induction of selected stress proteins at physiological temperatures in K562 and B16-F10 cells [10,11]. Of note, although benzyl alcohol and the close analogue phenethyl alcohol equally fluidized the PM, the latter did not reorganize the microdomains and subsequent HSP induction was absent, suggesting that a distinct reorganization of these microdomains is involved in the generation and transmission of stress signals to downstream HSP activation [10,12].

Different classes of PM microdomains have been recognized. Lipid rafts are PM microdomains enriched in cholesterol and sphingolipids which play an important role in the initiation of many signaling pathways. The fast, dynamic modulation of their structure results in an ever-changing content of both lipids and proteins which are essential for signal perception and transduction [13]. Caveolae are cholesterol-rich PM invaginations which cluster multiple proteins involved in signal transduction. Caveolae formation is regulated by the integral membrane protein caveolin-1 which is necessary for and governs the major functions attributed to caveolae through interaction with caveolae-localized proteins. Upon stimulation, caveolae pinch off from the PM and translocate to the cytoplasm where they act as intracellular regulators of signaling cascades [14].

How changes in plasma membrane physical properties during physiological stress are transmitted intracellularly is not completely understood. A redistribution of cholesterol rich lipid rafts in parallel with an increased packing density of PM lipids correlated with enhanced HSP expression levels following heat exposure [15]. Since the structure of those lipid rafts strongly depends on lipid-phase behavior, thermally-controlled changes in PM fluidity modify the lateral segregation behavior of the embedded domains further suggesting their involvement in heat sensing and initiation of HSR [10,16]. In addition, heat-induced translocation of caveolin from the caveolae to the perinucleus has been reported indicating the involvement of caveolae in heat stress sensing [17]. Although not exactly understood, cytosolic release of caveolae-contained proteins, including caveolin-1, could thus link the PM to downstream pathways through direct interaction with specific targets [14].

As cholesterol is critical for the formation and configuration of lipid rafts and caveolae, targeted modulations of PM cholesterol levels or mobility is a widely used tool to disrupt the dynamic character of those microdomains and to study their involvement in cellular physiology. Methyl-β-cyclodextrin (MβCD)—a cyclic polysaccharide with high affinity for cholesterol—is one of the most commonly used tools to extract cholesterol from cellular membranes [18]. Nystatin—a polyene sterol-binding antimycotic—has current therapeutic applications and operates through a bi-phasic concentration-depended mode of action in fungal ergosterol-containing membranes. At low concentrations, sterol sequestration (immobilization) is observed perturbing the lipid packing characteristics of the membrane and reducing the ability of cholesterol to interact with and exert its effects on other membrane components. With increasing nystatin concentrations, additional nystatin-oligomerization-induced pore formation is generally observed. In mammalian cholesterol-containing membranes, sterol sequestration was equally observed; however, even at higher concentrations, pore formation was absent [19].

In the current study, we aimed to compare the effect of MβCD- and nystatin-induced cholesterol modulations on heat-induced activation of HSP70 and HSP25—both known to be induced upon heat—in mouse B16-F10 melanoma cells. Next, we analyzed the effect of MβCD-induced cholesterol depletion on acquired thermotolerance, an adapted survival against extreme heat. With image-based fluorescence correlation spectroscopy, we analyzed the lateral diffusion constant of fluorescently labeled cholesterol in PM during MβCD and nystatin treatment. Finally, as MβCD actively extracts cholesterol out of PM, pushing the cells towards a new equilibrium, we performed in-depth lipidomics to follow the immediate effect of cholesterol deprivation on the whole cell lipidome.

## 2. Materials and Methods

### 2.1. Cyclodextrin and Nystatin

For each experiment, solutions of MβCD (CylcoLabs, Budapest, Hungary; 10 mM in serum-free RPMI medium) and nystastin (Sigma-Aldrich, Budapest, Hungary; 50 mg/mL in dimethyl sulfoxide (DMSO)) were freshly prepared. The MβCD used was a statistically methylated beta-cyclodextrin with an average degree of methylation of 1.8 methyl groups per glucopyranose units (altogether 12.6 methyl groups per cyclodextrin ring).

### 2.2. Cell Culture

Mouse B16-F10 (ATCC CRL-6475) melanoma cells were grown in RPMI medium supplemented with 10% fetal calf serum, 4 mM L-glutamine, and streptomycin/penicillin at 37 °C in humidified incubator with 5% CO_2_.

### 2.3. Analysis of HSP70 and HSP25 Expression Levels

Cells were either exposed to 10 mM MβCD for 10 min [18] or to 50 µg/mL nystatin for 1 h [20] at 37 °C followed by heat stress for the indicated time at 42 °C in serum-supplemented RPMI medium and 3 h recovery at 37 °C. Cells were lysed in RIPA buffer and HSP70 (ADI-SPA-810, Enzo Life Sciences, Farmingdale, NY, USA), HSP25 (ADI-SPA-801, Enzo Life Sciences), and GAPDH (G9545, Sigma-Aldrich) protein levels were analyzed through western blotting with the indicated antibodies. Signals were visualized by the use of HRP-conjugated secondary antibodies.

### 2.4. Analysis of HSF1 Expression/Posttranslational Modification Levels

Cells were either exposed to 10 mM MβCD for 10 min [18] or to 50 µg/mL nystatin for 1 h [20] at 37 °C followed by heat stress for the indicated time at 42 °C in serum-supplemented RPMI medium. Immediately after heat stress, cells were lysed in RIPA buffer and HSF1 (ADI-SPA-901, Enzo Life Sciences; RT-405, Thermo-Scientific, Waltham, MA, USA) and GAPDH (G9545, Sigma-Aldrich) were analyzed through western blotting with the indicated antibodies. Signals were visualized by the use of HRP-conjugated secondary antibodies.

### 2.5. Analysis of Cholesterol Levels

B16-F10 cells were exposed for 10 min to 10 mM MβCD at 37 °C followed by 30, 60, or 90 min heat stress at 42 °C in serum-supplemented RPMI medium. Immediately after stress, cholesterol levels were measured with the Amplex-red cholesterol assay kit according to the supplier’s guidelines (Thermo Scientific).

### 2.6. Stress Survival Experiments

For acquired thermotolerance (ATT) measurements, B16-F10 cells were exposed to 10 mM MβCD for 10 min at 37 °C followed by 30, 60, or 90 min heat stress at 42 °C in serum-supplemented RPMI medium. After 16–18 h recovery at 37 °C, all cells were exposed for 30 min to 45 °C. To estimate the fraction of surviving cells, resazurin, a fluorescent indicator of cellular metabolism, was added to the medium and cells were further incubated at 37 °C. At regular time points, the resulting change in fluorescence of growth medium was monitored at 565 nm excitation wavelength and 580 nm emission wavelength.

To test if a limited cholesterol resupply after MβCD treatment during heat stress exposure should have an effect on the survival of MβCD-pretreated cells, B16-F10 cells were incubated for 10 min with 10 mM MβCD at 37 °C followed by 60 or 90 min heat shock at 42 °C in serum-supplemented RPMI- or serum-free RPMI medium. After pre-exposure heat stress, serum-free medium was exchanged for complete medium and cells were allowed to recover for 16–18 h at 37 °C. Then, cells were exposed for 30 min at 45 °C and the following day, the fraction of surviving cells was estimated with resazurin as described above.

### 2.7. Image-Based Fluorescence Correlation Spectroscopy (ImFCS)

Cells were seeded into glass bottom dishes (MatTek Corporation, Ashland, MA, USA) two days before experiment. Measurements were performed in culturing media without phenol red at room temperature after labeling cells with 100 nM Abberior Star 488 PEG cholesterol (ASP-Chol; Abberior, Göttingen, Germany) for 5 min and the subsequent washing steps. Objective type Total Internal Reflection illumination was used for achieving the thinnest excited sample volume with a high numerical aperture objective (alpha Plan-FLUAR 100; Zeiss, Oberkochen, Germany). Excitation wavelength 488 nm from a Spectra-Physics Stabile 2018 (Spectra-Physics; Santa Clara, CA, USA) laser as light source was introduced to the microscope (Zeiss Axiovert 200) by two tilting mirrors. The laser beam was focused on the back focal plane of the objective after a 488 nm cleanup filter and aZT488/647/780rpc-UF1 dichroic mirror (Chroma Technology GmbH, Olching, Germany). Sample signal was collected by the objective and filtered by a 535/70 emission filter (Chroma). For acquisition, we used a ProEM512 EMCCD camera (Princeton Instruments, Trenton, NJ, USA) with 3 milliseconds effective exposure time and 20 × 40 pixel acquisition area per measurement (pixel size 0.16 µm). The image-based fluorescence correlation spectroscopy (ImFCS) plugin (http://www.dbs.nus.edu.sg/lab/BFL/imfcs_image_j_plugin.html) for ImageJ software was used for data evaluation. The autocorrelation functions (ACFs) for every pixels were calculated using a multi-tau correlation scheme [21]. An exponential of polynomial bleach correction was used to correct data before fitting. For obtaining the diffusion coefficient (D) for all pixels, ACFs were fitted as described earlier [22]. The decreased number of reporter molecules caused by cholesterol depletion does not affect the calculated D since this evaluated parameter is independent in a broad range of molecule number [23].

### 2.8. Lipidomics

B16-F10 cells were exposed to 10 mM MβCD for 2, 5, and 10 min at 37 °C and immediately thereafter collected. The pellets were shaken in 1 mL methanol containing 0.001% butylated hydroxytoluene as an antioxidant for 10 min and centrifuged at 10,000× *g* for 5 min. The supernatant was transferred into a new reaction tube and stored at −20 °C [24]. All experiments were done in two biological repeats, each containing three technical repeats. Mass spectrometry analyses were done as described earlier [25]. PLS-DA was performed with the Metaboanalyst suite 4.0 [26].

### 2.9. Statistics

Band intensities of HSP70 and HSP25 measured upon MβCD followed by heat were analyzed with 2-way ANOVA followed by Sidak’s multiple comparisons test. Band intensities of HSP70 and HSP25 upon nystatin followed by heat were analyzed with one-way ANOVA followed by Tukey’s multiple comparisons test. Band intensities of HSF1 upon MβCD or nystatin followed by heat were analyzed with one-way ANOVA followed by Tukey’s multiple comparisons test. Data of ATT experiments, effect of serum-supplemented versus serum-free medium experiments, and cholesterol replenishment experiments was analyzed with ANOVA followed by Tukey’s multiple comparisons test. 

## 3. Results

### 3.1. Plasma Membrane Modulations with Methyl-β-Cyclodextrin (MβCD) and Nystatin Impair the Heat-Induced Stress Response

Considering the involvement of cholesterol-rich PM microdomains in HSR, we wanted to compare the effect of MβCD- and nystatin-induced cholesterol modulations on the stress-induced activation of selected HSPs. We decided to focus on stress-induced activation of HSP70 (HSPA1A) and the small HSP HSP25 (HSPB1), representative HSPs known to be highly upregulated upon heat exposure.

**Figure 1:**
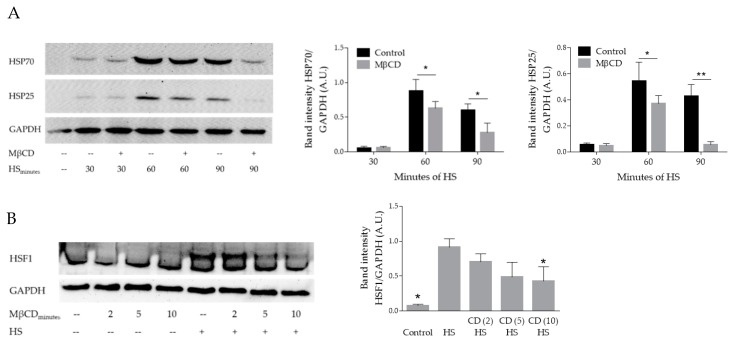

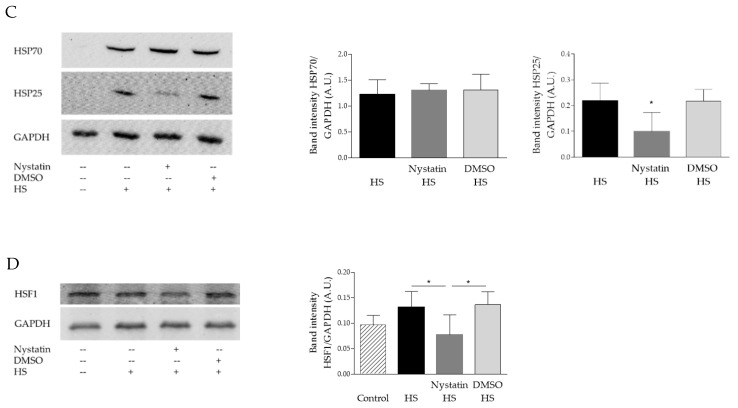
Effect of PM modulation on heat-induced heat shock response. (**A**) B16-F10 cells were incubated for 10 min with 10 mM Methyl-β-cyclodextrin (MβCD) at 37 °C followed by 30, 60, or 90 min of heat stress at 42 °C and 3 h recovery at 37 °C; (**B**) B16-F10 cells were incubated for 2, 5, or 10 min with 10 mM MβCD at 37 °C followed by 1 h heat shock at 42 °C; (**C**) B16-F10 cells were exposed for 1 h to 50 µg/mL nystatin at 37 °C followed by 1 h heat stress at 42 °C and 3 h recovery at 37 °C; (**D**) B16-F10 cells were exposed for 1 h to 50 µg/mL nystatin at 37 °C followed by heat stress for 1 h at 42 °C. Bar graphs show quantified band intensities normalized to GAPDH (*n* = 3), * *p* < 0.05; ** *p* < 0.01. CD (X): cells exposed for 2, 5, or 10 min to MβCD. DMSO was treated as a vehicle control for nystatin. A.U.: arbitrary units, HS: heat shock.

First, B16-F10 cells were incubated for 10 min with 10 mM MβCD at 37 °C followed by 30, 60, or 90 min heat stress at 42 °C and 3 h recovery at 37 °C. Compared to untreated cells, MβCD treatment resulted in lower heat-induced HSP70 and HSP25 levels in a time-dependent manner (Figure 1A). Considering the impaired heat-induced stress response upon MβCD exposure, we analyzed HSF1 post-translational modification (PTM) levels. Upon stress, HSF1 is modulated by multiple posttranslational modifications. Currently, 30 amino acids have been identified in the HSF1 sequence which are susceptible to phosphorylation, acetylation, summoylation, and O-glycosylation [4]. As PTMs add to the molecular weight of the targeted protein, this might result in a pronounced band shift which can be visualized by western blotting. B16-F10 cells exposed for 2, 5 or 10 min to 10 mM MβCD at 37 °C followed by 1 h heat shock at 42 °C had a reduced HSF1 band shift in a time-dependent manner (Figure 1B), suggesting a modulated HSF1 posttranslational profile.

Next, B16-F10 cells were exposed for 1 h to 50 µg/mL nystatin at 37 °C followed by 1 h heat stress at 42 °C and 3 h recovery at 37 °C. Compared to untreated cells, nystatin resulted in reduced heat-induced HSP25 levels but had no effect on HSP70 levels (Figure 1C). We then analyzed for nystatin-induced changes in HSF1 expression/post-translational modification levels and exposed B16-F10 cells for 1 h to 50 µg/mL nystatin at 37 °C followed by heat stress for 1 h at 42 °C. Immediately after heat stress, nystatin exposure resulted in a less pronounced HSF1 signal compared to the heat stress control (Figure 1D).

### 3.2. Plasma Membrane Modulation with MβCD Alters Acquired Thermotolerance of B16-F10 Cells

Acquired thermotolerance (ATT) is an adapted ability of cells to survive otherwise lethal heat in response to an earlier pre-exposure to non-lethal stress. Considering the observed effects of targeted PM modulation by MβCD on heat-induced HSP70 and HSP25 expression, we next wanted to analyze its effect on ATT.

**Figure 2:**
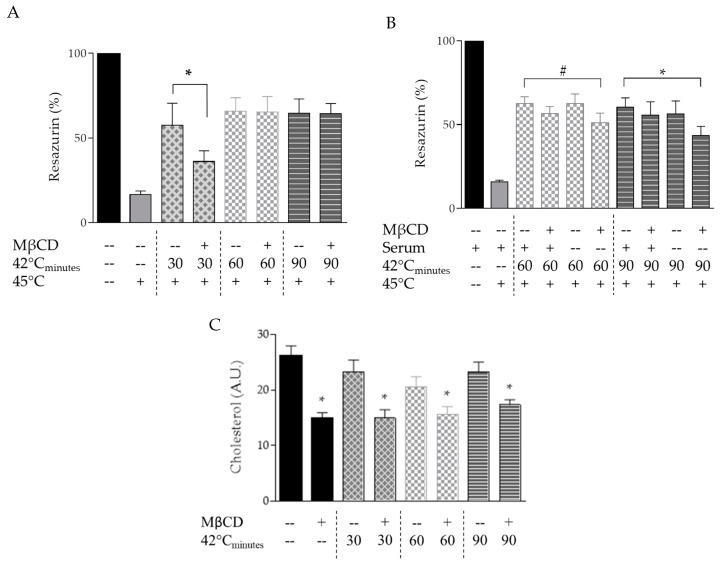
Effect of MβCD-induced PM modulation on development of acquired thermotolerance (ATT) in B16-F10 cells. (**A**) B16-F10 cells were exposed for 10 min to 10 mM MβCD at 37 °C followed by 30, 60, or 90 min of heat stress at 42 °C (pre-exposure). Then, after 16–18 h recovery at 37 °C, cells were re-exposed to heat stress for 30 min at 45 °C. The following day, the fraction of surviving cells was estimated with resazurin (*n* = 4, * *p* < 0.05); (**B**) B16-F10 cells were incubated for 10 min with 10 mM MβCD at 37 °C followed by 60 or 90 min heat shock at 42 °C in serum-supplemented- or serum-free RPMI medium (limiting cholesterol supply). After pre-exposure heat stress, serum-free medium was exchanged for complete medium and cells were allowed to recover for 16–18 h at 37 °C. Then, cells were exposed for 30 min at 45 °C and the following day, the fraction of surviving cells was estimated with resazurin (*n* = 3, ^#^ *p* = 0.06, * *p* < 0.05); (**C**) B16-F10 cells were exposed for 10 min to 10 mM MβCD at 37 °C followed by 30, 60, or 90 min heat shock at 42 °C and cholesterol levels were measured immediately after heat stress (*n* = 6, * *p* < 0.05 compared to 37 °C control,). A.U.: arbitrary units.

First, B16-F10 cells were exposed for 10 min to 10 mM MβCD at 37 °C followed by 30, 60, or 90 min of heat stress at 42 °C in serum-supplemented RPMI medium (pre-exposure). Then, after 16–18 h recovery at 37 °C, cells were re-exposed to heat stress for 30 min at 45 °C. The following day, the fraction of surviving cells was estimated with resazurin. Upon 30 min of pre-exposure heat, MβCD resulted in reduced ATT compared to non-treated heat-shocked cells. However, from 60 min pre-exposure heat onwards, a similar ATT was observed in MβCD-treated cells compared to the time-matched control cells (Figure 2A).

Following this observation and considering the very high affinity of MβCD for cholesterol, we hypothesized that while being exposed to heat stress, plasma membrane cholesterol levels might re-equilibrate allowing for restored heat sensing and resulting stress response as observed from 60 min pre-exposure heat onwards. Thus, a limited cholesterol resupply after MβCD treatment during heat stress exposure should have an effect on the survival of MβCD-pretreated cells. To test this possibility, B16-F10 cells were incubated for 10 min with 10 mM MβCD at 37 °C followed by 60 or 90 min heat shock at 42 °C in serum-supplemented RPMI- or serum-free RPMI medium. After pre-exposure heat stress, serum-free medium was exchanged for complete medium and cells were allowed to recover for 16–18 h at 37 °C. Then, cells were exposed for 30 min at 45 °C and the following day, the fraction of surviving cells was estimated with resazurin. As expected, pre-exposure heat in serum-supplemented RPMI medium for 60 min or longer resulted in similar ATT in MβCD-treated cells compared to time-matched pre-exposure heat control cells (Figure 2B). However, pre-exposure heat in serum-free RPMI medium for 60 min almost reached statistical significantly lower ATT in MβCD-treated cells compared to time-matched pre-exposure heat control cells (*p* = 0.06), whereas pre-exposure heat in serum-free RPMI medium for 90 min resulted in significantly lower ATT in MβCD-treated cells compared to time-matched pre-exposure heat control cells (*p* < 0.05) (Figure 2B). Considering the high affinity of MβCD towards cholesterol, this suggested that most probably cholesterol supply from the serum-supplemented medium influences the restoration of the MβCD-impaired stress response.

Thus, we analyzed cholesterol levels during pre-exposure heat in MβCD-treated cells. B16-F10 cells were exposed for 10 min to 10 mM MβCD at 37 °C followed by 30, 60, or 90 min heat shock at 42 °C and cholesterol levels were measured immediately after heat stress. At every time point measured, cholesterol levels of MβCD-treated cells did not recover to baseline and were always significantly lower compared to those of untreated non-heat-shocked control cells (Figure 2C). This would suggest that apart from cholesterol, hitherto unknown factors present in serum might influence the restoration of the MβCD-impaired stress response.

Treatment of B16-F10 cells with nystatin resulted in reduced heat-induced HSP25 expression level (Figure 1C). Considering the published involvement of HSP25 on development of thermotolerance [27,28], we did not perform nystatin-related ATT assays as a similar effect as to that observed with MβCD was anticipated.

### 3.3. Exposure to MβCD Alters the Lateral Diffusion of Cholesterol in the Plasma Membrane

**Figure 3:**
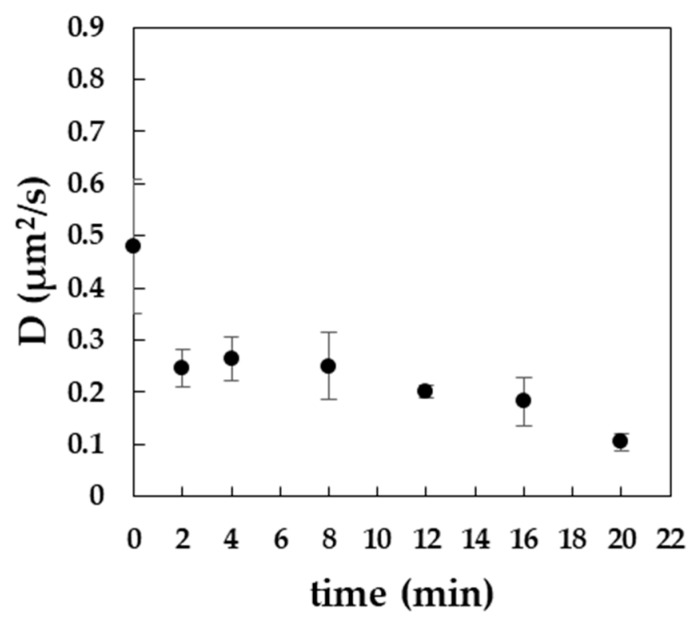
Diffusion constant of Abberior Star 488 PEG cholesterol probe (ASP-Chol) in the plasma membrane of B16-F10 cells upon exposure to 10 mM MβCD for the indicated time.

To analyze the immediate effect of PM modulation on the lateral diffusion of cholesterol, a fluorescent analogue (ASP-Chol) was used which reports only from the outer leaflet of PM since the flip/flop of this reporter is prevented due to the polyethylene linker. First, imaging fluorescence correlation spectroscopy (ImFCS) measurements were performed on labeled cells before and during MβCD exposure. The significant decrease of lateral diffusion speed observed within the first minutes of MβCD exposure was followed by a slower decay (Figure 3). Next, ImFCS measurements were performed on labeled cells before and after 1 h of nystatin exposure. However, exposure to nystatin did not result in any significant change of the diffusion constant (data not shown).

### 3.4. Lipidomics Analysis Indicates an Immediate and Extensive MβCD-Induced Lipidome Remodeling

As MβCD actively extracts cholesterol out of PM, we anticipated that it might push the cell towards a new PM compositional equilibrium. Thus, we decided to perform in-depth lipidomics to follow the immediate effect of MβCD-induced cholesterol deprivation on the whole cell lipidome.

**Figure 4:**
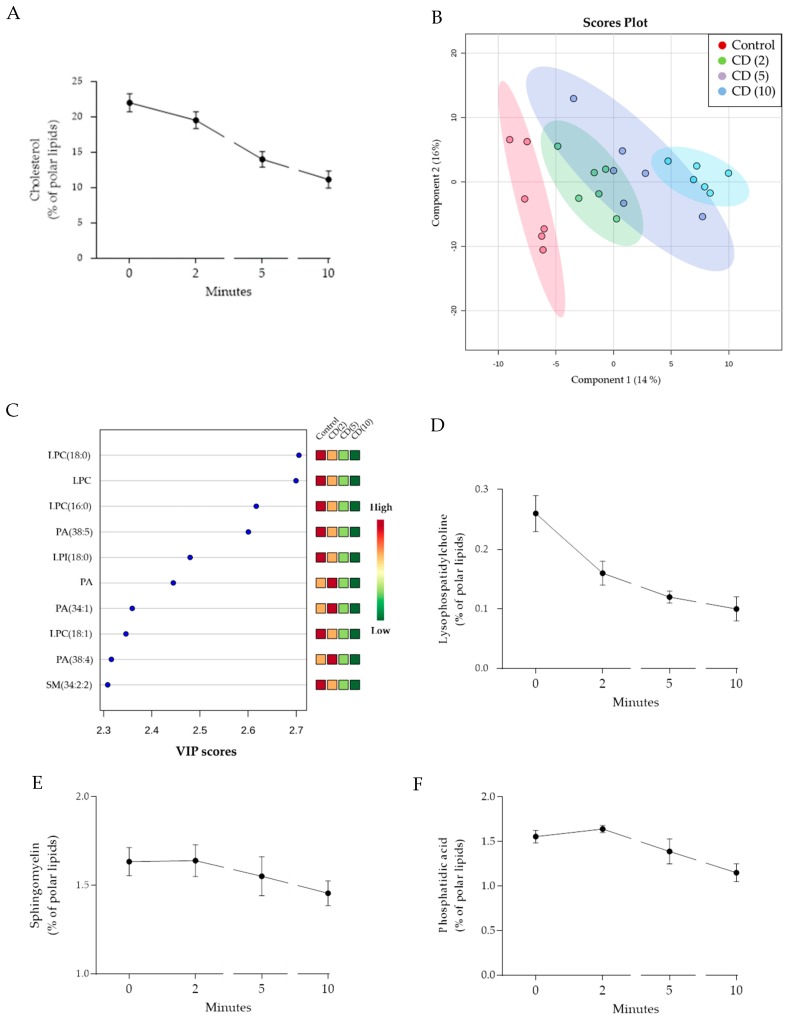
Effect of MβCD on B16-F10 lipidome. (**A**) Time-dependent changes in the levels of cholesterol; (**B**) PLS-DA scores plot generated without cholesterol values; (**C**) Most important discriminative features due to MβCD treatment: lysophosphatidylcholine (LPC), phosphatidic acid (PA), lysophosphatidylinositol (LPI), sphingomyelin (SM); Time-dependent changes in the levels of lysophosphatidylcholine (**D**), sphingomyelin (**E**), and phosphatidic acid (**F**). CD (X): cells exposed for 2, 5, or 10 min to MβCD.

B16-F10 cells were treated with 10 mM MβCD for 2, 5, and 10 min at 37 °C and immediately thereafter the total cellular lipid content was isolated and analyzed with mass spectrometry. First, we confirmed the established affinity of MβCD towards cholesterol in our dataset by reaching a reduction of up to 50% of cholesterol levels in 10 min (Figure 4A). Next, before proceeding with the statistical analysis, we removed the cholesterol values from our dataset. As such, we avoid that the large effect of MβCD on cholesterol might mask more subtle but potentially relevant changes of other lipid species. Interestingly, PLS-DA analysis [29] still indicated a good separation of the different time points (Figure 4B); cross-validation with 2 components revealed good predictability (Q2 = 0.74) and high goodness of fit (R2 = 0.93). Sphingomyelin, lysophosphatidylcholine, and phosphatidic acid species were identified among the 10 mostly altered features due to MβCD exposure (Figure 4C). By analyzing the time-dependent changes in the levels of these lipid species, an immediate decrease of lysophosphatidylcholine species was visible (Figure 4D) whereas phosphatidic acid and sphingomyelin species displayed a more delayed onset of decrease (Figure 4E,F).

In mammalian cholesterol-containing membranes, nystatin operates through sterol sequestration/immobilization without the formation of pores [19]. Therefore, in-depth nystatin-related lipidomics were not performed as similar cholesterol levels compared to control were anticipated excluding the need for the cells to reach a new PM compositional equilibrium.

## 4. Discussion

The involvement of cholesterol-rich PM microdomains such as lipid rafts and caveolae in HSR was previously suggested [17,30]. To further study the involvement of those microdomains in HSR, we compared the effect of MβCD- and nystatin-induced modulations on stress-induced activation of the representative HSPs HSP70 and HSP25 in mouse B16-F10 melanoma cells.

Treatment with MβCD resulted in diminished heat-induced HSP70 and HSP25 expression (Figure 1A), whereas treatment with nystatin diminished heat-induced HSP25 expression without affecting HSP70 induction (Figure 1C). These observations suggest that, under the conditions used, MβCD and nystatin likely acted upon different cholesterol pools. Interestingly, a specific selectivity of MβCD-induced cholesterol depletion towards lipid raft regions has been suggested, depending on exposure time (up to 10 min) and/or concentration used [18]. Additionally, a specific disruption of caveolae by nystatin without modifying other PM microdomains was previously suggested by deep-etch freeze microscopy [20]. Consequently, based on our results and considering the published conditional selectivity of MβCD and nystatin towards respectively lipid rafts and caveolae, it is tempting to speculate that selective heat-induced activation of lipid rafts or caveolae are likely to target specific HSP subpopulations. Consistent with our nystatin data is the intriguing observation that genetic disruption of caveolin-1—an essential component of functional caveolae—in mouse mammary tumor cells was shown to impair the expression of HSP25 but not HSP70 [31].

In line with the observed altered HSP profile compared to untreated heat shocked cells, we observed an altered stress-induced HSF1 PTM pattern upon PM modulation with MβCD or nystatin (Figure 1B,D). Upon heat, HSF1 is targeted by multiple PM-originating signaling cascades which play a defining role in its activation [32]. Currently, we can only speculate which signaling cascade(s) might be affected. For example, both JNK [33] and p38 MAP kinases [34] were previously suggested to take part in membrane-associated HSP25 induction. Of note, cholesterol depletion inhibited JNK and p38 MAP kinase-associated signaling in different model systems [35,36]. Thus, it is tempting to speculate that MβCD- or nystatin-induced PM modifications impair different signaling cascades towards HSF1 resulting in an altered PTM profile. Although the precise role of HSF1 PTMs is unknown [37] it is suggested to provide specificity towards its binding preferences to selected heat shock elements in the promotor region of a subset of hsp genes [38]. In addition, induction of HSP70 and HSP25 depends on the nuclear domain ND-10-associated proteins Daxx and PML [39]. Previously, interaction of Daxx and HSF1 during HSR was reported [40]. In fact, in mouse embryonic fibroblasts, release of Daxx from the nuclear domain correlated with HSP25 suppression whereas release of PML correlated with lower HSP70 levels. Considering that Daxx acts as a regulator of cholesterol synthesis through association with the androgen receptor [41], it is tempting to speculate that MβCD-induced cholesterol depletion could titrate Daxx away from the HSF1 regulatory complex resulting in the observed impaired HSP25 induction upon heat.

Interestingly, MβCD impaired acquired thermotolerance in B16-F10 when cells were pre-exposed to a short (30 min) period of sublethal heat (Figure 2A). However, prolonged pre-exposure (>60 min) to sublethal heat did not result in MβCD-induced impaired acquired thermotolerance. Multiple scenarios might explain this observation. (a) MβCD-induced alterations in PM cholesterol levels and microdomain disruption are restored during prolonged pre-exposure to sublethal heat resulting in restored signaling cascades and HSP expression levels. The finding that even prolonged pre-exposure to sublethal heat in serum-free medium—limiting external cholesterol supply—impaired acquired thermotolerance, supports this assumption (Figure 2B). However, even when cells were pre-exposed for up to 90 min of sublethal heat in serum-supplemented medium, cellular cholesterol levels were not restored to levels comparable to those of untreated cells (Figure 2C). Additionally, prolonged pre-exposure for up to 90 min to sublethal heat did not result in restored HSP expression levels (Figure 1A). (b) As a potential feedback mechanism, prolonged pre-exposure to sublethal heat might activate alternative signaling and/or survival mechanisms resulting in restored survival. In fact, the mouse macrophage tumor cell line P388D1 displayed heat-induced thermotolerance in the absence of HSF1 transactivation capacity and subsequent HSP induction [42]. Additionally, CHO [25] and murine B-cell lymphoma CH1 [43] cells displayed heat-induced thermotolerance in the complete absence of HSP expression. As of now, we can only speculate about the potential nature of these alternative mechanisms. Currently, in-depth RNAseq experiments are ongoing in our lab to explore the specific underlying molecular mechanisms responsible for the observed restored ATT upon prolonged pre-exposure to sublethal heat.

Recently, by using advanced fluorescence imaging and spectroscopy approaches, a two-component diffusion model for cholesterol in the PM of live cells was proposed suggesting a heterogeneous diffusion in the cell membrane which is due to its nanoscale interactions and localization in the membrane [44]. In the current study, by using a fluorescently-labeled cholesterol analogue as a reporter for lateral cholesterol diffusion, we observed a quick decrease of the diffusion constant during MβCD treatment (Figure 3). Although our fluorescent cholesterol probe might not completely reflect the native behavior of the endogenous cholesterol, this might suggest an altered composition and structure of the PM caused by the cholesterol depletion. Of note, one-hour nystatin exposure did not affect the lateral diffusion of the cholesterol probe. Currently, we can only speculate about the differences in the observed changes in lateral diffusion between MβCD- or nystatin exposure and assume that they might be due to their respective mode of action (extraction vs. sequestration/immobilization).

Apart from cholesterol, we identified additional potentially relevant MβCD-induced changes in the lipidome of B16-F10 cells. In addition to previously reported MβCD-induced decreases in sphingomyelin levels [18], a gradual decrease in lysophosphatidylcholine (LPC) and phosphatidic acid (PA) species was observed (Figure 4D–F). Thus, our study indicates that MβCD affects other lipid species as well suggesting that the effects of MβCD on cell physiology as described in the literature might go well beyond changes in cholesterol levels and in fact be of a much more complex nature. Precisely how these changes came about—active uptake by MβCD, lipid metabolism, or passive leakage/active transport into the extracellular milieu—is currently not known.

Of note, based on in vitro studies, a chaperone-like function of LPC able to prevent thermally-induced protein denaturation was suggested, implying a potential function in preserving the conformation and function of PM-embedded signaling proteins during heat stress [45]. On the other hand, PA functions as a precursor for the generation of bioactive lipids such as diacylglycerol (DAG) [46]. Intriguingly, since perception of heat stress at the level of PM relies among others on DAG-mediated arachidonic acid generation which ultimately modulates HSF1 activity [32], these minor but relevant reduction in LPC and/or PA levels might also have a potential role in the observed impaired stress sensing. In fact, we earlier demonstrated the role of nutritional lipid supply to cell culture medium in stress-sensing through reorganization of cholesterol-rich microdomains [47]. Currently, we can only speculate about the potential underlying mechanisms of these changed lipid species levels in our findings which should be addressed in future studies. For example, the size and/or function of the specific lipid-associated HSP70 pools—as recently discussed by Balogi et al. [48]—could be affected by the observed MβCD-induced lipidome alterations and might be of importance when interpreting our current findings.

## 5. Conclusions

Our data demonstrated impaired heat-induced HSP expression levels upon targeted PM modulation in B16-F10 cells. These data not only highlight the involvement of PM integrity in HSR but also suggest that altered dynamics of specific cholesterol pools could represent a mechanism to fine tune HSP expression. Considering that cholesterol exchange between cells through direct cell–cell contacts has recently been shown [49], membrane-compound exchange through cell-to-cell communication could in fact represent an hitherto less recognized mechanism through which stress adaptation could spread throughout a larger cell population.
